# The complete chloroplast genome of *Sarcandra glabra* (Chloranthaceae): a perianthless basal angiosperm

**DOI:** 10.1080/23802359.2018.1473722

**Published:** 2018-06-06

**Authors:** Eun-Kyeong Han, Won-Bum Cho, Goya Choi, Jung-Hyun Lee

**Affiliations:** a Department of Biology Education, Chonnam National University, Gwangju, Republic of Korea;; b Herbal Medicine Research Center, Korea Institute of Oriental Medicine, Daejeon, Republic of Korea

**Keywords:** Chloroplast genome, phylogenetic analysis, *Sarcandra glabra*

## Abstract

We have obtained the complete chloroplast (cp) genome sequence for *Sarcandra glabra*. This genome is 158,881 bp long, with 39.2% GC content. It includes a large single copy region of 88,169 bp that is separated from the 18,446-bp small single copy region by two inverted repeat regions (26,133 bp each). This genome contains 130 genes, i.e. 85 protein-coding genes, 37 tRNA, and eight tRNA. Maximum likelihood analysis, based on 13 complete cp genomes, showed that *S. glabra* is closely related to two other family members, *Chloranthus spicatus* and *C. japonicus*.

*Sarcandra glabra* (Thunb.) Nakai is an evergreen shrub belonging to Chloranthaceae. Although these plants are widely distributed in the tropical to subtropical vegetation zones of Asia (Xia and Jérémie 1999), this species is extremely rare on Jeju Island (Korea). There are many plants that show unique evolutionary characteristics (Lee et al. 2014). As its population size continues to decrease due to human activity, plants on Jeju Island are being managed as endangered organisms (National Institute of Biological Resources 2012). Only two plastid genomes have been sequenced within Chloranthaceae (further Chloranthales), and they are regarded as early-diverging lineages of angiosperms (Soltis et al. 2005). Therefore, our research results can serve as a fundamental genetic source for designing markers for conservation at the genetic level. Our findings can also be used in phylogenomics approaches to uncover evolutionary clues for early-diverging angiosperm species.

Fresh leaves of *S. glabra* were collected from Benoki, Kunigami-gun, Okinawa, Japan (*N* 26° 47′ 09.2″, E 128° 15′ 41.3″). The specimen was stored in the herbarium at the Korea Institute of Oriental Medicine (KIOM: YSG_KIOM-2015-61). Total genomic DNA was extracted with a DNeasy Plant Mini Kit (Qiagen, Seoul, Korea) and then sequenced on the Illumina Mi-Seq platform (LAS, Seoul, Korea). This generated 12,611,114 paired-end reads (2 × 300 bp). After quality assessment, clean reads of *S. glabra* were mapped with the chloroplast (cp) genomes of two other members of Chloranthales – *Chloranthus spicatus* (EF380352) and *C. japonicus* (KP256024), using Geneious 10.2.3 (Kearse et al. 2012). Annotations were done with the DOGMA program (Wyman et al. 2004) and tRNAscan-SE (Schattner et al. 2005). The annotated cp genome sequence was deposited in GenBank (accession no. MH0717440). We then conducted a phylogenetic analysis of the complete cpDNAs from 13 species and included *Amborella trichopoda* as an outgroup. In all, 74 protein-coding genes were aligned with ClustalW (Thompson et al. 1994). Maximum likelihood analysis was performed using RAxML v8.2.11 (Stamatakis 2014) with 1000 bootstrap replicates and the GTR GAMMA model.

The entire cp genome for *Sarcandra glabra* is 158,881 bp long and its GC content is 39.2%. It contains a large single copy region of 88,169 bp, a small single copy region of 18,446 bp, and a pair of inverted repeat regions that are 26,133 bp each. The genome encodes 130 genes, including 85 protein-coding genes, 37 tRNA, and eight rRNA. Its gene content and order are quite similar to that of *Chloranthus* spp. (Sun et al. 2016). Phylogenetic analysis of 13 cp genomes indicated that *S. glabra* forms a monophyletic group with other species of Chloranthales that are sister to Magnoliids (Figure 1). Therefore, this complete cp genome provides basic genetic information for future phylogenetic and conservation studies.

**Figure 1.**
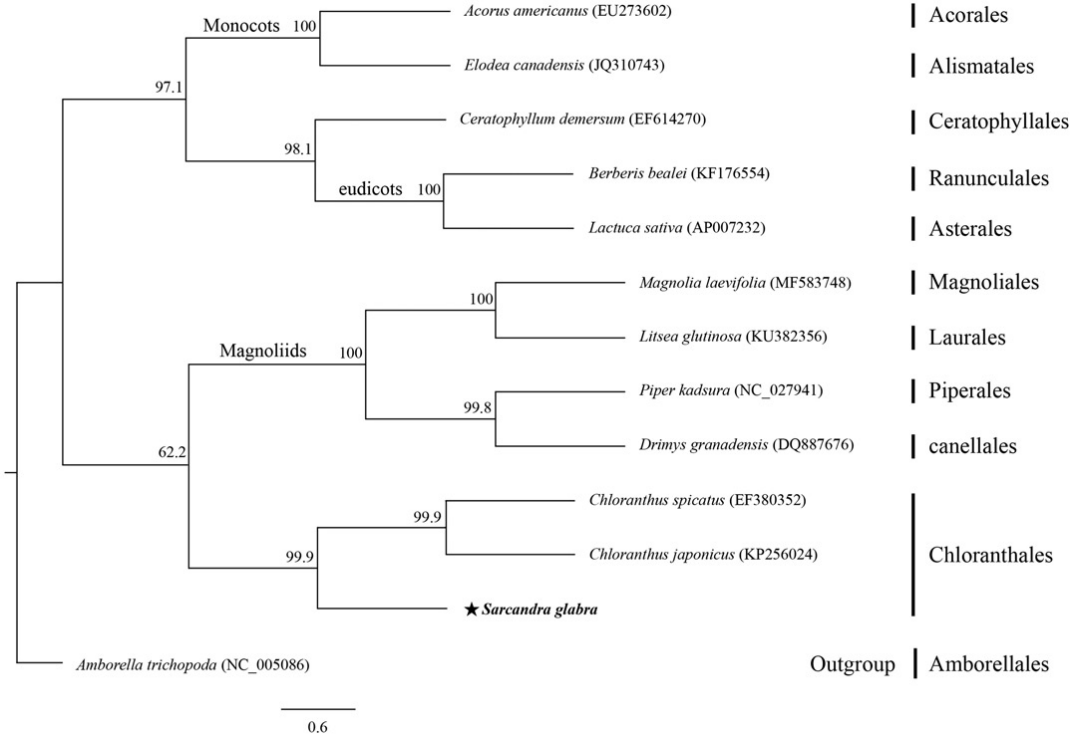
Maximum-likelihood tree based on 74 common protein-coding genes from 13 complete chloroplast genomes. *Amborella trichopoda* (Amborellales) was used as outgroup. Bootstrap values are shown next to nodes.
